# Autoantibodies in Autoimmune Hepatitis: Can Epitopes Tell Us about the Etiology of the Disease?

**DOI:** 10.3389/fimmu.2018.00163

**Published:** 2018-02-16

**Authors:** Urs Christen, Edith Hintermann

**Affiliations:** ^1^Pharmazentrum Frankfurt/ZAFES, Goethe University Hospital, Frankfurt am Main, Germany

**Keywords:** LKM-1, cytochrome P450 2D6 epitopes, epitope mapping, epitope spreading, molecular mimicry, diagnostic antibodies, pathogenic antibodies

## Abstract

Autoimmune hepatitis (AIH), primary biliary cholangitis (PBC), and primary sclerosing cholangitis (PSC) are serious autoimmune liver diseases that are characterized by a progressive destruction of the liver parenchyma and/or the hepatic bile ducts and the development of chronic fibrosis. Left untreated autoimmune liver diseases are often life-threatening, and patients require a liver transplantation to survive. Thus, an early and reliable diagnosis is paramount for the initiation of a proper therapy with immunosuppressive and/or anticholelithic drugs. Besides the analysis of liver biopsies and serum markers indicating liver damage, the screening for specific autoantibodies is an indispensable tool for the diagnosis of autoimmune liver diseases. Such liver autoantigen-specific antibodies might be involved in the disease pathogenesis, and their epitope specificity may give some insight into the etiology of the disease. Here, we will mainly focus on the generation and specificity of autoantibodies in AIH patients. In addition, we will review data from animal models that aim toward a better understanding of the origins and pathogenicity of such autoantibodies.

## Introduction: Autoimmune Liver Diseases

There are three major autoimmune diseases that target the liver. These autoimmune liver diseases affect either the liver parenchyma like in autoimmune hepatitis (AIH) or the bile ducts like in primary biliary cholangitis (PBC) and PSC. They all have in common that an aggressive autoimmune reaction results in destruction of liver tissue, which may subsequently ensue the development of severe hepatic fibrosis.

Autoimmune hepatitis is an often life-threatening disease characterized by the progressive destruction of the parenchyma and the development of chronic fibrosis (1–5). AIH occurs in children and adults of all ages, has a female predominance (sex ratio, 3.6:1), and affects different ethnic groups with an overall prevalence of 10–20 cases per million persons in Northern Europe and the United States (6–8). The disease is primarily associated with the presence of HLA class I B8 and HLA class II DR3, DR4, and DR52a (9–11). The histological hallmark of AIH is the presence of interface hepatitis, characterized by piecemeal necrosis affecting patches of hepatocytes (3, 12). In addition, according to the revised and simplified scoring system of the International AIH Group (IAIHG) (13), one of the core diagnostic criteria of AIH and its subtypes is the presence of specific antibodies to particular liver autoantigens (see below). Besides antibodies and histology, the IAIHG scoring system also considers hypergammaglobulinemia and the absence of viral markers (13). The current standard therapy of AIH is a glucocorticoid treatment with prednisone or prednisolone alone or in combination with azathioprine (14). However, recently alternative treatments have been successfully introduced, in particular for patients suffering from AIH relapses after corticosteroid withdrawal. The next generation glucocorticoid budesonide (15) as well as the calcineurin inhibitors cyclosporine A and tacrolimus potentially could improve the outcome of AIH (16, 17). Interestingly, the combination treatment budesonide/azathioprine resulted in fewer side effects than the conventional prednisone/azathioprine therapy in AIH patients without cirrhosis (15). However, the clinical guidelines of the European Association for the Study of the Liver (EASL) does not recommend using budesonide in patients with cirrhosis or peri-hepatic shunting, since the lack of efficient first-pass hepatic clearing of budesonide might result in undesired side effects (14). In addition, the immunosuppressant mycophenolate mofetil (MMF), a cytostatic drug that reversibly inhibits the purine biosynthesis, has been demonstrated to be safe and effective as first-line or rescue therapy in inducing and maintaining remission (18). EASL clinical practice guidelines suggest using MMF mainly as a second-line therapy in cases of azathioprine intolerance (14). Unfortunately, during standard therapy, adults rarely achieve resolution of their laboratory and liver tissue abnormalities in less than 12 months, and withdrawal of therapy after 2 years leads to relapse in 85% of cases (6). Moreover, these long-term therapies carry the risk of significant steroid-specific and azathioprine-related side effects.

PBC, formerly known as primary biliary cirrhosis, has recently been renamed to primary biliary cholangitis due to a lack of consistent cirrhosis in a large proportion of patients (19). It has an incidence ranging from 0.3 to 5.8 in 100,000 and has clear female dominance (F:M 9:1) (20). PBC is an autoimmune liver disease characterized by a chronic cholestasis, destruction of the intrahepatic small bile ducts, and the presence of anti-mitochondrial antibodies (AMA) in over 95% of patients (21–23). The target structure is cholangiocytes/biliary epithelial cells (BEC) that are attacked by an aggressive autoimmune response occurring due to a loss of tolerance against several liver autoantigens, including the E2-subunits of the pyruvate dehydrogenase complex (PDC-E2), branched chain 2-oxo acid dehydrogenase, and 2-oxo-glutarate dehydrogenase (22). It has been shown that AMA contribute to the pathogenesis of PBC by increasing macrophage-derived TNFα production resulting in enhanced apoptosis of BEC (24). Besides AMA, particular antinuclear antibodies (ANA) specific for the nuclear body-associated protein sp100 or the nuclear pore membrane protein gp120 are present in more than 50% of PBC patients (25). Although alternative treatments are being evaluated, current therapy is still largely restricted to the administration of ursodeoxycholic acid (UDCA) (26). However, almost 40% of patients are unresponsive to UDCA treatment (27). Recently, obeticholic acid in combination with UDCA has been approved as the first new drug in almost 20 years for treatment of PBC, especially of patient refractory to UDCA single treatment (28, 29). Alternative unlicensed drugs include the corticosteroid budesonide as well as fibric acid derivatives, which act *via* activation of peroxisome proliferator-activated receptors (PPARs). However, there is yet no clear evidence that a therapy with budesonide or fibrates alone or in combination with UDCA is superior to UDCA monotherapy (30).

Finally, PSC is a chronic cholangiopathy characterized by progressive inflammation of the bile duct region resulting in the development of biliary fibrosis, which can advance to cirrhosis and hepatobiliary malignancy (31). PSC has an annual incidence of approximately 1 in 100,000 (32), is typically diagnosed between 30 and 40 years of age, and has a male predominance (M:F 2:1). Most PSC patients display damage of the large bile ducts (90–95%) with characteristic strictures and dilatations of the biliary tree as well as onion skin fibrosis surrounding the damaged ducts. About 20% of patients show small bile duct damage that progresses to large duct disease over a period of 10 years (33). Strikingly, approximately 70–80% of PSC patients also present with inflammatory bowel disease (IBD) and are associated with a higher risk for malignancies (34). Patients with PSC do not generate AMA, but a significant proportion of patients generate “atypical” perinuclear anti-neutrophil cytoplasmic antibodies (pANCA). However, such antibodies are not considered for diagnostic purposes (35). Patients suffering from PSC have a higher risk for hepatobiliary malignancies, but even among PSCpatients with cirrhosis the risk for developing a hepatocellular carcinoma is low (36). In contrast to PBC, the administration of UDCA is controversial for the therapy of PSC. A meta-analysis of several clinical trials revealed no beneficial role of UDCA in slowing the progression of PSC (37). Alternative treatments including the UDCA derivative NorUDCA and agonists to several nuclear receptors, such as farnesoid X receptor and PPAR, are under current investigation in preclinical models (31). Besides PBC and PSC, immunoglobulin G4-associated cholangitis (IAC) is another biliary disease that presents with biochemical and cholangiographic features that are very similar to those found in patients with PSC (38). IAC is characterized by elevated serum immunoglobulin G4 (IgG4) levels and marked infiltration of liver and bile ducts by IgG4-positive plasma cells and contrary to PSC, IAC is not associated with IBD (38). The EASL clinical practice guidelines suggest a corticosteroid as an initial treatment followed by azathioprine in patients with proximal and intrahepatic stenoses and/or relapses during/after corticosteroid therapy.

In addition to the three major autoimmune liver diseases, several overlap syndromes (OS) have been described. According to IAIHG, patients are classified as having an OS if they display overlapping features within the spectrum of AIH and PBC or AIH and PSC (39). OS are not rare occurrences, since a considerable proportion of AIH patients also exhibit features of PBC (7–13%), PSC (6–11%), or a cholestatic syndrome with additional diagnostic features, such as specific antibodies (5–11%) (40). For diagnosis of the AIH-PBC OS the so-called “Paris criteria” have been suggested (41). They include PBC criteria, such as elevated serum levels exceeding the upper limit of normal values by at least a factor 2 for alkaline phosphatase (AP) and a factor of 5 for γ-glutamyl transpeptidase (GGT), presence of AMA, and a liver biopsy showing bile duct lesions. On the AIH side, the criteria comprise serum levels of alanine aminotransferase (ALT) that are elevated by at least five times the upper limit of normal values, serum levels of immunoglobulin G (IgG) that are at least two times higher than the upper limit of normal values, presence of AIH-typical autoantibodies, and a liver biopsy showing interface hepatitis with moderate or severe periportal or periseptical lymphocytic piecemeal necrosis (41). These criteria have been verified in a larger study with 134 PBC, AIH, or AIH-PBC OS patients confirming a high level of sensitivity and specificity for the detection of an AIH-PBC OS (42). AIH-PSC OS is histologically characterized by the presence of an interface hepatitis with or without the presence of plasma cells, portal edema or fibrosis, ductopenia, ductal distortion, ductular proliferation, cholate stasis or, in some patients, obliterative fibrous cholangitis (40). By cholangiography, focal strictures and dilatations of the biliary tree characteristic for PSC are often found in patients with diagnosed AIH, resulting in diagnosis of AIH-PSC OS instead (40). In addition, the criteria for AIH-PSC OS include elevated levels of AST/ALT, γ-globulin, IgG, AP, GGT as well as the absence of AMA that would point toward PBC (40).

## Autoantibodies in AIH

Historically, three types of AIH have been classified upon the presence of specific autoantibodies. In type 1 AIH (AIH-1) ANA and/or SMA are typical, whereas type 1 liver/kidney microsomal antibodies (LKM-1) have been considered as the hallmark of type 2 AIH (AIH-2). In addition, the term type 3 AIH (AIH-3) has been used to classify patients with antibodies directed against soluble liver antigen (SLA) (3, 6, 12, 43). However, recently such a classification has been questioned since patients with AIH-1 and AIH-2 share the same clinical phenotype (44). Due to the observed change of the autoantibody profile from one subtype to another in some patients over time, AIH-2 might as well constitute an early form of AIH appearing in younger patients who later during disease convert to a AIH-1. In addition, AIH-3 is considered obsolete since anti-SLA autoantibodies are often present together with other antibodies that point toward AIH-1 (45). In this review, we will adhere to the traditional classification into AIH-1 and AIH-2. Possibly the most complex group of autoantibodies are the ANA. In patients with AIH-1 the target structure of ANA in the nucleus is the entire chromatin, including DNA, centromers, histones, sn-RNPs, and cyclin A (46, 47), whereas in PBC ANA are more specifically reacting to histones and centromers, respectively. Anti-centromer antibodies (ACA) are found in up to 30% of PBC patients, who mostly also suffer from systemic sclerosis (SSc) for which ACA are considered as a diagnostic marker (48). Approximately 80% of patients with a PBC/SSc overlap syndrome carry ACA (48, 49). However, ANA are also found in patients with drug-induced hepatitis, chronic hepatitis B or C, as well as in patients with non-alcoholic fatty liver disease (NAFLD) (50). There is not much known about how NAFLD is influencing AIH, but many patients with non-alcoholic steatohepatitis also manifested signs of AIH, including interface hepatitis and ANA generation (51). Interestingly, experimental AIH is exacerbated in mice with NAFLD (52). The precise target molecules for many ANA have not yet been identified. Thus, the actual pattern of nuclear staining is important for diagnosis and the mere presence of any ANA may be compatible with AIH-1 but is not considered a *bona fide* diagnostic marker. To achieve diagnostic value, a detailed analysis of the staining patterns and a consideration of the actual ANA titers is required (50). Similarly, SMA recognizing filamentous actin are valid as diagnostic antibodies for AIH-1 if evaluated carefully. Like ANA, SMA can be detected in other liver diseases with an autoimmune or viral background, but the titers are normally higher in AIH-1. In addition, the staining pattern of SMA on rat kidney sections is mainly focused on tubular and glomerular structures (53). More detailed information on staining patterns of ANA and SMA including images of immunocytochemistry and immunohistochemistry is available in recent review articles by Liberal et al. (50) and Muratori et al. (53).

In patients with AIH-2, the target for anti-LKM-1 antibodies has been identified as the 2D6 isoform of the large cytochrome P450 enzyme family [cytochrome P450 2D6 (CYP2D6)] (54, 55). Anti-LKM-1 antibodies are considered diagnostic, if a hepatitis C virus (HCV) infection can be excluded, since reactivity to CYP2D6 has also been found in chronic hepatitis C patients (see Molecular Mimicry and Epitope Spreading) (56–58). Besides CYP2D6, two additional targets recognized by LKM-1 antibodies have been identified as ERp57 and carboxylesterase 1 (CES1) (59). Although ANA, SMA, or LKM-1 are the most frequent autoantibodies generated in patients with AIH, some patients have no detectable or only marginal titers. However, they may carry other autoantibodies such as peripheral antinuclear neutrophil antibodies that have also been termed “atypical” pANCA since they recognize in contrast to “typical” pANCA beta-tubulin isotype 5, rather than myeloperoxidase (60). Further autoantibodies include anti-SLA and anti-liver and pancreas antigen (LP) antibodies both recognizing UGA suppressor tRNA-associated protein (61), liver cytosol type 1 antibodies (LC-1) specific for formiminotransferase cyclodeaminase (FTCD) or type 3 liver/kidney microsomal antibodies (LKM-3) recognizing family 1 UDP glucuronosyltransferases (3). Like LKM-1, LC-1 antibodies are considered *bona fide* diagnostic markers for AIH-2, whereas LKM-3 have only a minor significance in AIH diagnosis, since they have also been detected in a fraction of patients with hepatitis D (62, 63) and have only a low sensitivity (3, 53). LKM-2 antibodies recognizing cytochrome P450 2C9 have been reported in some patients with AIH-1 or AIH-2 but are predominantly associated with drug-induced hepatitis induced by tienilic acid (64, 65). Furthermore, anti-liver-specific membrane lipoprotein (LSP) antibodies and, reacting to asialoglycoprotein receptor (ASGPR), which is highly expressed at the surface of hepatocytes, are present in up to 88% of patients (66) and may be used as a general marker compatible with AIH-1 or AIH-2, but not as a diagnostic tool, since they are found also in patients with other liver diseases, such as chronic hepatitis B and C, alcoholic liver disease, and PBC (67). Anti-liver membrane antibodies, which show also reactivity to ASGPR are less well defined and are rarely used. Recently, the reactivity to ASGPR in sera of patients with different autoimmune liver diseases has been investigated using an improved ELISA (68). It has been found that 29.1 and 16.7% of patients with AIH-1 and AIH-2, respectively, carry autoantibodies against ASGPR. However, using the same method such autoantibodies have also been found in patients with PSC or hepatitis C (68).

There is a plethora of commercial kits for autoantibody detection available, some of which use obsolete or outdated autoantibody and/or target antigen nomenclature. Thus, it is important to keep in mind that the conventional markers for AIH-1 are ANA and SMA, whereas LKM-1 are the hallmark autoantibodies used for diagnosis of AIH-2. A summary of autoantibodies in AIH is displayed in Table 1. Besides serving as disease markers or even as *bona fide* diagnostics tools autoantibodies might also be involved in the pathogenesis of AIH. Interestingly, the presence of anti-SLA antibodies has been associated with a more severe phenotype of AIH (69). Thus, one possibility would be that anti-SLA antibodies might actively enhance the hepatocellular damage. However, such additional autoantibodies might also originate as result of enhanced hepatocellular destruction as the associated release and presentation of critical amounts of additional liver autoantigens can drive the expansion of SLA-specific B cells. Quite a while ago, it has been found that the titers of LSP antibodies reacting to ASGPR correlated with the severity of AIH (70). However, again a higher titer might just be the result of an exacerbated state of disease, rather than an indication of a pathogenic nature of the antibody. In addition, LSP antibodies are not specific for AIH.

**Table 1:** Autoantibodies in autoimmune hepatitis.

	Autoantibody	Target structure/molecule	Diagnostic value
Type 1 AIH (AIH-1)	Antinuclear antibodies	Chromatin	Yes, after detailed analysis of staining pattern in immunocytochemistry
	Anti-smooth muscle antibodies	Filamentous actin; tubular and glomerular specificity in kidney	Yes, after detailed analysis of staining pattern in immunocytochemistry
	Soluble liver antigen/LP	UGA suppressor tRNA-associated protein	Associated with severe phenotype
	Type 2 liver/kidney microsomal antibodies	Cytochrome P450 2C9	No, more associated with drug-induced hepatitis
	Peripheral antinuclear neutrophil antibodies	Beta-tubulin isotype 5	Compatible with AIH-1
Type 2 AIH (AIH-2)	Type 1 liver/kidney microsomal antibodies	Cytochrome P450 2D6	Yes, if hepatitis C virus is excluded
	Type 2 liver/kidney microsomal antibodies	Cytochrome P450 2C9	No, more associated with drug-induced hepatitis
	Type 3 liver/kidney microsomal antibodies	Family 1 UDP-glucuronosyltransferases	Yes, but low sensitivity
	Liver cytosol type 1 antibodies	Formiminotransferase cyclodeaminase	Yes
	Liver-specific membrane lipoprotein	Asialoglycoprotein receptor	Compatible with AIH-2

Mechanistically, antibodies might be involved in the pathogenesis of AIH by decorating hepatocytes and thereby induce complement-mediated cell lysis. Indeed, antibodies have been detected at the surface of hepatocytes isolated from liver biopsies (71). Interestingly, CYP2D6, one of the main target autoantigen of LKM-1 antibodies, has been initially found to be expressed at the surface of rat hepatocytes and therefore might have been indeed an excellent target for LKM-1 antibodies (72). However, subsequent more detailed studies could not confirm this finding (73, 74). The observations that cellular infiltrations detected in interface hepatitis are dominated by CD4, rather than CD8 T cells or other lymphoid cells (75) and that most serum autoantibodies are of the IgG isotype (76) might indicate that CD4 T cells execute an essential helper function in the pathogenesis of AIH. In summary, so far there is no firm evidence for the presence of pathogenic autoantibodies in AIH. In animal models, the presence of autoantibodies to liver autoantigens FTCD or CYP2D6 is not sufficient to induce AIH-like disease (77, 78). Thereby, an immunization of mice with recombinant CYP2D6 resulted in the generation of anti-CYP2D6 antibodies, but no substantial T cell response in the liver and no clinical features of AIH (77). In addition, transfer of total IgG isolated from mice with AIH-like disease and high titers of anti-CYP2D6 antibodies (>1/10,000) did not induce AIH-like disease in naïve recipient mice (Holdener and Christen, unpublished data). Furthermore, Hardtke-Wolenski et al. recently demonstrated that the genetic background does not play a role in the generation of autoantibodies to CYP2D6 or FTCD but is important for the development of AIH. Administration of FTCD encoded by a liver-specific virus resulted in AIH-like disease in the autoimmunity-prone non-obese diabetic mice, but not in normal Balb/c, C57BL/6, or FVB/N mice (78). Further, they found that the generation of autoantibodies was required, but not sufficient, for the development of AIH (78). In summary, although there is no firm proof for a direct pathogenic effect, it is likely that autoantibodies are more than just clinical markers and contribute at least partially to the chronic inflammation of the liver.

## CYP2D6 Epitopes

**Figure 1:**
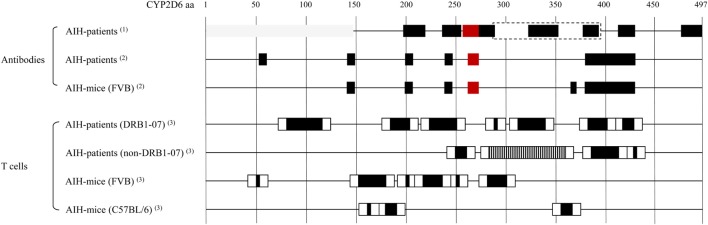
Cytochrome P450 2D6 (CYP2D6)-specific antibody and T cell epitopes: B cell/antibody and T cell epitopes detected in patients with type 1 AIH (AIH-1) and in the CYP2D6 mouse model. Note that B cell/antibody epitopes are similar in patients and mice, whereas due to differences in the MHC, the T cell epitopes are different. Red boxes: immunodominant epitope recognized by high titer autoantibodies in most of autoimmune hepatitis (AIH) patients. Gray boxes: conformational epitopes (87, 88). Dashed box: large epitope with no further subdivision, possibly dominated by the indicated smaller epitopes within (87). T cell epitope mapping has been performed using staggered, overlapping 20-mer peptides covering the entire CYP2D6 protein, therefore the epitope sequences have been divided into a core (back boxes) and peripheral (white boxes) region. Vertical striped box: several overlapping epitopes (91). (1) Collective data from Ref. (82, 73, 79–80, 85–88). (2) Patient and mouse data from the same epitope mapping assay (84). (3) Data from epitope mapping using the same set of staggered 20-mer CYP2D6 peptides (77, 91).

The major autoantigen in AIH-2, CYP2D6, is the best characterized autoantigen in AIH. Extensive epitope mapping has been performed in patients as well as in mouse models (Figure 1). Early after the identification of CYP2D6 as the target of LKM-1 antibodies, an immunodominant B-cell epitope has been mapped to a region spanning amino acids 254–271 (aa254–271). This epitope has been recognized by sera of the majority of patients with AIH-2, ranging from 62 to 100%, depending on the individual study (79–84). Besides this immunodominant epitope, several other regions of CYP2D6 have been identified as molecular targets for LKM-1 antibodies in various proportions of patients’ sera, including the sequential regions spanning aa321–351, aa373–389, and aa410–429 (82); aa196–218 (85); aa193–212, aa238–257, aa268–287, and aa478–497 (86); aa55–63, aa139–147, aa203–211, aa239–aa247, and aa379–aa429 (84), aa284–391, aa412–429, as well as conformational epitopes located in the region of aa1–146 (87) and aa321–379 (88). The majority of these epitopes is located at the surface of the CYP2D6 molecule and is therefore easily accessible to autoantibodies (84).

The CYP2D6 molecule is also recognized by CD4 and CD8 T cells if properly presented by MHC I or II. Such autoreactive CYP2D6-specific CD4 and CD8 T cells have been found in the blood and the liver of patients with AIH-2 (89, 90). By testing the proliferative T cell response to 61 overlapping peptides covering the entire CYP2D6 molecule, a polyclonal reactivity to seven regions has been found in HLA DRB1*07 and four regions in non-DRB1*07 patients (Figure 1) (91). Furthermore, by using HLA-A2–CYP2D6–peptide tetramers, CYP2D6-specific CD8 T cells producing high levels of IFNγ have been found in AIH-2 patients. Whereby IFNγ production and cytotoxicity were higher at the time of diagnosis than after beginning of immunosuppressive treatment, and the frequency of CYP2D6-specific CD8 T cells correlated well with the severity of the disease (92).

## Molecular Mimicry and Epitope Spreading

One possibility of how the reactivity of autoantibodies might give insight into the initiation and/or propagation of AIH is a concept known as “molecular mimicry” (93–96). Thereby, a similarity between a pathogen component and a self-antigen would cause the pathogen-specific antibodies and/or T cells to attack the similar self-antigen as well [see Ref. (97) for a detailed review on molecular mimicry]. Pathogen infections might play a role as drivers of an autoimmune process on several other levels, including causing direct damage to hepatocytes and triggering a strong inflammatory response in the liver (98). However, evidence for such triggering pathogen infections is hard to find, since the pathogen itself might have disappeared at the time of diagnosis (hit-and-run event) and only susceptible individuals develop autoimmune manifestations. In addition, more than just one trigger might be necessary to induce an autoimmune disease, and it has been demonstrated that some pathogens might even prevent autoimmunity by either deleting autoaggressive lymphocytes (99) or by inducing counteracting suppressive mechanisms, i.e., regulatory T cells (100).

**Figure 2:**
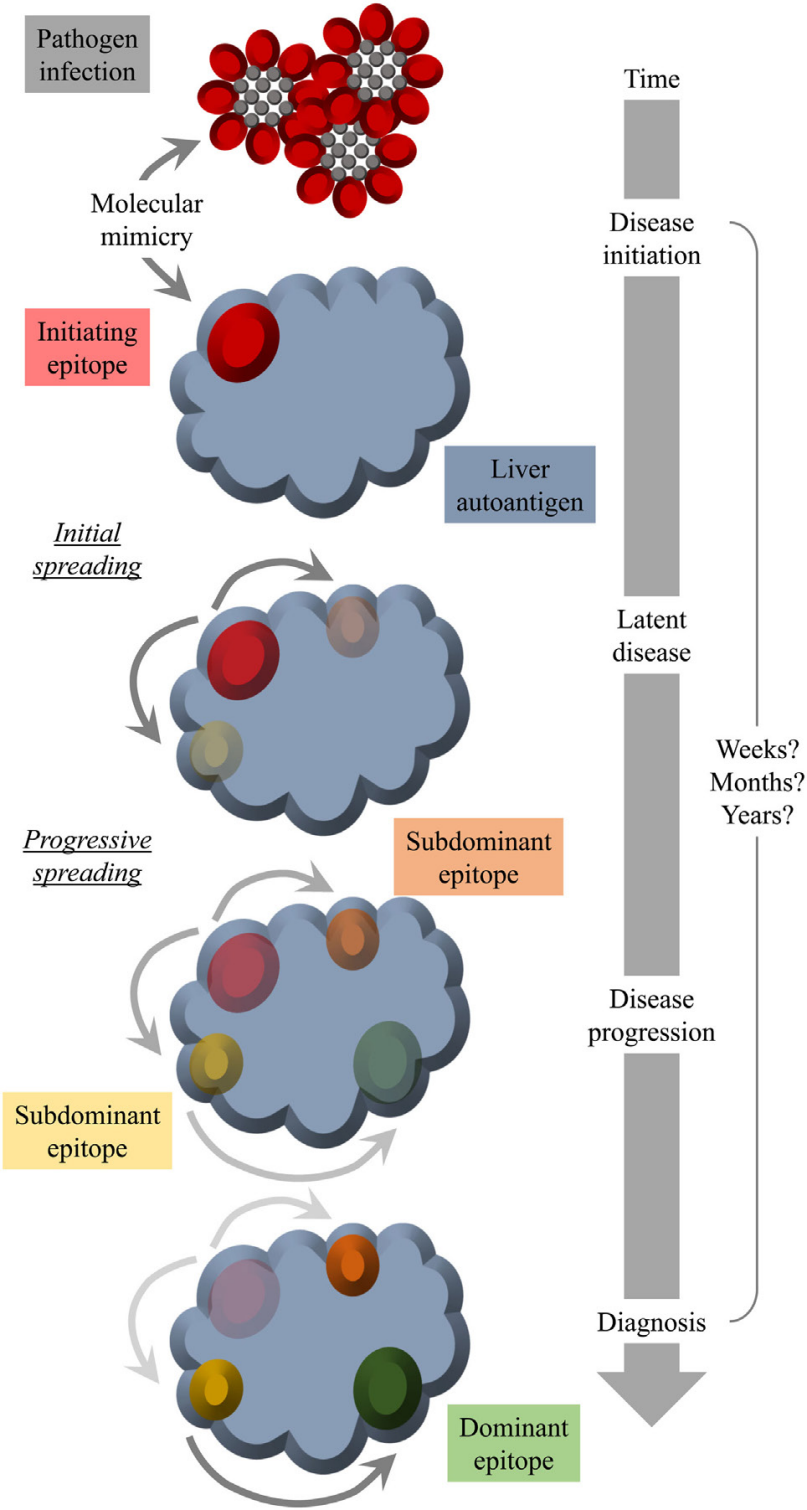
Molecular mimicry and epitope spreading: hypothetical scenario for environmental triggering factors as inducers of autoimmune disease. Infection of the host by a pathogen that shares a structural similarity with a host molecule (molecular mimicry) occurs long before diagnosis. The initial epitope recognized by specific antibodies functions as an origin of intramolecular epitope spreading occurring as result of somatic hypermutations in B cells and the subsequent dynamic antibody response. Thereby, the reactivity to the initiating epitope might be lost over time leaving behind immunodominant epitopes that have nothing in common with the pathogen structure that was responsible for the initiation of the autoreactivity. Thus, at the time of diagnosis, there is no obvious link to an infection with a pathogen that mimics a host component.

Another factor that hampers the detection of a possible structural similarity between pathogen and self-antigen is a mechanism termed “epitope spreading” or “determinant spreading” (101). Thereby, the initial immune response (antibodies and/or T cells), which is directed against the initiating epitope, would spread intramolecularly to other epitopes of the same self-antigen and in some instances even intermolecularly to other self-antigens. Since the initiating epitope might not necessarily be the final immunodominant one, the specificity of the patients’ autoantibodies and/or T cells might be strongest to a late epitope appearing as result of spreading and not to the initiating epitope at time of diagnosis. A possible scenario for an involvement of molecular mimicry as well as epitope spreading is displayed in Figure 2.

Unfortunately, such a scenario is difficult to demonstrate in patients, since processes of molecular mimicry and epitope spreading might have long passed at the time of diagnosis. In PBC and in other autoimmune diseases, including type 1 diabetes, multiple sclerosis, rheumatoid arthritis, and autoimmune Addison’s disease, the development of autoantibodies may precede the clinical onset of disease by 10 years or even more (102). Indeed, epitope mapping with sera collected from AIH patients over more than a decade after diagnosis revealed a very stable epitope specificity (84). This indicates that in most patients only a steady state rather than a dynamically developing immune specificity can be observed. Therefore, we have developed an inducible mouse model for AIH using adenovirus encoded human CYP2D6 (Ad-2D6) as a trigger. Infection of wild-type C57BL/6 or FVB mice with Ad-2D6 results in the breakdown of tolerance to the mouse Cyp homologs that are similar, but not identical, to the human CYP2D6 (83, 103). Such Ad-2D6-infected mice develop AIH-like disease characterized by cellular infiltrations with an interface hepatitis-like pattern, hepatic fibrosis, and the generation of CYP2D6-specific autoantibodies (83) and T cells (77). Several animal models for AIH have been developed in the past [see Ref. (104) for a detailed review on current models]. However, the presence of a clearly defined target autoantigen and the possibility of being able to initiate the autoimmune response/disease at a defined time allow the use of the CYP2D6 model to perform a detailed study of the CYP2D6-specific immune response over time. Thus, the hypothesis that a pathogen infection might be involved in the etiology of AIH can be evaluated.

We found that Ad-2D6-infected mice first develop antibodies reactive to the region around the CYP2D6 sequence DPAQPPRD (aa263–270), indicating that this region might be involved in the initiation of the CYP2D6-specific immune response (84). At later times after infection, the antibody reactivity spreads to other epitopes, which are predominantly located at the surface of the CYP2D6 molecule (84). A similar predominance of surface epitopes has also been previously reported using sera of patients with AIH-2 (105). Importantly, even 8 weeks after infection, the highest titers of anti-CYP2D6 antibodies were determined for the initiating DPAQPPRD (aa263–270) epitope (84). Thus, in the mouse model the initiating epitope remains immunodominant over time. Interestingly, this epitope is also immunodominant in patients with AIH-2 (Figure 2). This stands in contrast to T cell epitopes, which are found to be dissimilar in patients and mice (77, 91). Naturally, the difference in MHC molecules able to present critical CYP2D6 peptides predominantly accounts for this divergence (Figure 1).

Several of the identified linear CYP2D6 B cell epitopes share sequence homologies to human pathogens. Already at the time of identification of the immunodominant CYP2D6 epitope DPAQPPRD (aa263–270), a shared sequence homology with the infected cell protein 4 of herpes simplex virus 1 (HSV-1) has been reported (81). Since this immunodominant region has been also detected by LKM-1 antibodies of (depending on the study) up to 100% of AIH patients (79) and has been identified as the initiating and immunodominant region in the CYP2D6 mouse (84) HSV-1 infection might indeed be involved in the etiology of AIH. Unfortunately, no epidemiological evidence supports such an association between AIH and HSV-1 infection. By contrast, there is epidemiological evidence for an association between HCV infection and the development of AIH-2 (106, 107). In addition, LKM-1 antibodies have been detected in up to 10% of patients with a chronic HCV infection (56–58). Interestingly, *vice versa* antibodies to HCV have been found in a large proportion of AIH-2 patients, which suggests that AIH patients might have experienced HCV infection in the past (108, 109). In fact, it has been demonstrated that antibodies specific for the HCV proteins NS3 and NS5a cross-react to a specific conformational epitope on CYP2D6 spanning aa254–288 (110), which contains the immunodominant epitope DPAQPPRD (aa263–270). Further screening of the NCBI GenBank revealed additional sequence homologies to the immunodominant CYP2D6 epitope by the envelope glycoprotein E1 of HCV and by proteins of the human immunodeficiency virus (HIV) (84). Several sequence homologies of subdominant CYP2D6 epitopes that have appeared later as the initiating epitope have been found to various human pathogens, including HCV, HIV, rabies virus, human cytomegalovirus, Karposi’s sacrcoma associated herpes virus, and *Legionella pneumophila*, and with several *Mycobacterium, Burkholdria*, and *Brucella* species (84).

Another triggering factor for autoimmune liver diseases might be protein adduct formation by reactive drug metabolites. The best documented case of drug-induced hepatotoxicity with an autoimmune component is halothane hepatitis (111–113). Upon oxidative, cytochrome P450 2E1 (CYP2E1)-dependent metabolism of the anesthetic agent halothane, trifluoroacteylated protein adducts (TFA-adducts) are formed, which act as neoantigens. Susceptible patients generate TFA-adduct-specific antibodies and T cells and develop a fulminant hepatitis. Several such neoantigens have been identified and include CYP2E1 as well as CYP2D6 (113). Interestingly, TFA-adduct-specific antibodies generated in patients and in experimental animals cross-react with the lipoic acid moiety of the E2-subunits of the 2-oxoacid dehydrogenase family enyzmes, including PDC-E2, which constitute the major autoantigens in PBC (114, 115). Furthermore, CES1, which has been identified as additional target autoantigen recognized by LKM-1 antibodies is also a target antigen in halothane hepatitis (116). However, sera of patients with AIH or halothane hepatitis react to different epitopes (59). The anesthetic agent halothane has been withdrawn from the market in the early 1990s; however, the closely related general anesthetic isoflurane is still in use. Protein modifications similar to TFA-adducts are also formed upon anesthesia with modern isoflurane derivatives, such as desflurane. In addition, some hydrochlorofluorocarbons (HCFC), which are frequently used as foam blowing agents, refrigerants, and propellants, are metabolized in a similar way, giving raise to TFA-adducts as well (117). Although cases of isoflurane (118), desflurane (119), and HCFC hepatitis (120) have been reported, there is yet no firm proof that drug-adduct formation and the subsequent generation of drug-adduct-specific antibodies contribute to the development of AIH or another autoimmune liver disease.

## Conclusion

The diagnosis of autoimmune liver diseases is difficult and relies on histological analysis of liver biopsies as well as systematic serology, including the presence of specific autoantibodies and distinct enzymatic makers that indicate the nature of the liver damage. Thus, a detailed characterization of autoantibody pattern and titer is indispensable for the diagnosis of AIH, as well as other autoimmune liver diseases, such as PBC and PSC. In fact, with proper analysis of immunofluorescent staining patterns and autoantibody titer a serologic reactivity is found in more than 95% of AIH patients (121). In AIH, some autoantibodies correlate with the severity of the disease, but there is no firm proof that such autoantibodies are pathogenic *per se*. In general, there is still desperate need for more knowledge on the etiology and immunopathogenesis of AIH to develop novel therapeutic interventions. AIH therapy still largely relies on a corticosteroid and/or cytostatic drug regimen and since autoimmune diseases are a lifelong burden, such chronic therapies are often associated with long-term side effects. Novel animal models (104) might provide a basis to identify crucial inflammatory factors that drive the disease pathogenesis and/or contribute to its chronicity.

## Author Contributions

All authors listed have made a substantial, direct, and intellectual contribution to the work and approved it for publication.

## Conflict of Interest Statement

The authors declare that the research was conducted in the absence of any commercial or financial relationships that could be construed as a potential conflict of interest.

## Abbreviations

AIH, autoimmune hepatitis; PBC, primary biliary cholangitis; PSC, primary sclerosing cholangitis; AMA, anti-mitochondrial antibodies; BEC, biliary epithelial cells; MMF, mycophenolate mofetil; PDC-E2, E2-subunits of the pyruvate dehydrogenase complex; BCOADC-E2, branched chain 2-oxo acid dehydrogenase; OGDC-E2, 2-oxo-glutarate dehydrogenase; UDCA, ursodeoxycholic acid; OCA, obeticholic acid; IBD, inflammatory bowel disease; FXR, farnesoid X receptor; PPAR, peroxisome proliferator-activated receptor; IAC, immunoglobulin G4-associated cholangitis; pANCA, perinuclear anti-neutrophil cytoplasmic antibodies; pANNA, peripheral antinuclear neutrophil antibodies; OS, overlap syndromes; AP, alkaline phosphatase; GGT, γ-glutamyl transpeptidase; ALT, alanine aminotransferase; IgG, immunoglobulin G; AIH-1, AIH-2, and AIH-3, type 1, type 2, and type 3 AIH, respectively; ANA, antinuclear antibodies; SMA, anti-smooth muscle antibodies; SLA, soluble liver antigen; ACA, anti-centromer antibodies; SSc, systemic sclerosis; NAFLD, non-alcoholic fatty liver disease; NASH, non-alcoholic steatohepatitis; LKM-1, type 1 liver/kidney microsomal antibodies; LKM-3, type 3 liver/kidney microsomal antibodies; CYP2D6, cytochrome P450 2D6; CYP2E1, cytochrome P450 2E1; LC-1, liver cytosol type 1 antibodies; FTCD, formiminotransferase cyclodeaminase; LSP, liver-specific membrane lipoprotein; ASGPR, asialoglycoprotein receptor; NOD, non-obese diabetic; aa, amino acid; Ad-2D6, adenovirus encoding human CYP2D6; ICP4, infected cell protein 4; HSV-1, herpes simplex virus 1; HCV, hepatitis C virus; HIV, human immunodeficiency virus; HHV-5, human cytomegalovirus; HHV-8, Karposi’s sacrcoma associated herpes virus; TFA-adduct, trifluoroacteylated protein adduct; HCFC, hydrochlorofluorocarbons.
